# Single-cell transcriptomics of Pacific white shrimp (*Litopenaeus vannamei*) hepatopancreas reveal immune and metabolic responses to AHPND-causing *Vibrio parahaemolyticus*

**DOI:** 10.3389/fimmu.2026.1713369

**Published:** 2026-01-27

**Authors:** Johanna E. Aldersey, Jason W. Abernathy, Miles D. Lange, Julio C. García, Craig A. Shoemaker, Benjamin H. Beck

**Affiliations:** 1 ARS Research Participation Program, Oak Ridge Institute for Science and Education (ORISE), Oak Ridge, TN, United States; 2 United States Department of Agriculture, Agricultural Research Service, Aquatic Animal Health Research Unit, Auburn, AL, United States

**Keywords:** acute hepatopancreatic necrosis disease, aquaculture, hepatopancreas, host-pathogen interaction, *Litopenaeus vannamei*, shrimp, single-cell transcriptomics, *Vibrio parahaemolyticus*

## Abstract

**Background:**

The shrimp aquaculture industry is severely impacted by acute hepatopancreatic necrosis disease (AHPND) caused by the bacterium *Vibrio parahaemolyticus*. The hepatopancreas is a multi-functional organ with roles in digestion, immunity, molting and reproduction. The mechanism by which the pathogen causes disease, and the host immune response is not completely understood. Therefore, we set out to characterize the cells of the hepatopancreas and host response to bacterial infection at single-cell resolution.

**Methods:**

First, hepatopancreas from three healthy Pacific white shrimp (*Litopenaeus vannamei*) were sampled to produce a single-cell transcriptomic atlas. Then, the hepatopancreas from three *V. parahaemolyticus* infected and two mocked treated shrimp were sampled for an infection study. Primary cell suspensions were produced, and single-cell libraries were generated using the 10x Genomics Chromium X controller with GEM-X 3’ gene expression reagents. Libraries were sequenced and data aligned to the shrimp reference genome using Cell Ranger. Seurat and clusterProfiler were used for downstream analyses.

**Results:**

The atlas consists of 11,006 quality cells that were grouped into nine clusters, and represent the hepatopancreas epithelial cells, myocytes and hemocytes. The infection study generated 16,368 quality cells and was integrated with the atlas for 27,374 cells grouped into nine clusters. Cells from the infected shrimp exhibited expression of immune related genes including diverse pathogen recognition receptors and humoral proteins, including hemocyanin, proteases and C-type lectins. We also found that cells expressed factors that the PirA/B toxins present in the infective *V. parahaemolyticus* may bind to, such as fatty acid binding protein (*Fabp*). In response to infection, energy metabolism (oxidative phosphorylation) was altered in a cluster-dependent manner which may reflect immune or pathogenic processes.

**Conclusions:**

We characterized the cells types of the hepatopancreas and examined the transcriptomic response to a virulent isolate of *V. parahaemolyticus*, the causative agent of AHPND. Cells exhibited significant humoral immune responses suggesting the role of these genes in immune responsiveness to the pathogen. The outcomes will inform future functional studies and provide insights toward novel preventative measures or treatments.

## 1 Introduction

Pacific white shrimp (*Litopenaeus vannamei*) topped the charts as the most produced aquaculture species worldwide in 2022 at 6.8 million tons (1). Despite the industry’s success, it is significantly challenged by infectious diseases such as acute hepatopancreatic necrosis disease (AHPND). The disease first appeared in China in 2009 (2). In the United States, AHPND was first reported on Texan *L. vannamei* farms after mass mortalities (3). The occurrence of the disease in Texas caused ~40% decrease in production from 296 tons in 2016 to 114 tons the following year (3). Mortalities caused by AHPND typically occur within five weeks of stocking a pond with post-larvae, and clinical signs include lethargy, soft shells, sinking to the bottom of the culture system, slow growth, pale hepatopancreas, and death. The hepatopancreas is the most impacted organ and, at a histological level, sloughing of the epithelial cells is observed. *Vibrio parahaemolyticus* harboring the pVA1 plasmid (VP_AHPND_) is the main causative agent of hepatopancreatic necrosis disease in shrimp (4).

The hepatopancreas is the major metabolic organ located in the cephalothorax of decapoda (5). The organ consists of two bilaterally symmetric tubular systems consisting of hundreds of blindly ending tubules. The hepatopancreas functions to digest food, absorb nutrients, and produce and store energy. The organ also expresses hemolymph proteins and aids vitellogenesis for ovary maturation and immunity (5). The epithelial cells of the tubules consist of E-cells (embryonic cells), R-cells (resorptive cells), F-cells (fibrillar cells), B-cells (blister-like cells) and M-cells (midget cells). Vogt (5) summarized the known function of these cells across decapod crustaceans though some are less characterized. E-cells are the progenitor cells that differentiate to R-, F- and B- cells. R-cells are the most abundant cell-type in the hepatopancreas and they absorb and metabolize nutrients, store energy and minerals, and synthesize lipoproteins and vitellogenin (6). F-cells synthesize common digestive enzymes, hemocyanin and immune defense molecules (7). The mature B-cell features a large central vacuole, which suggests that B-cells absorb material from the lumen (5). M-cells are thought to have an endocrine function that regulates the activity of hepatopancreatic cells or the muscular net encasing the organ to fill and empty the tubules (5). The interstitium between the tubules, called the hemolymph sinus, is filled with hemolymph supplied by the paired hepatopancreatic arteries (5).

VP_AHPND_ is associated with two major virulence factors, *Photorhabdus* insect-related toxins A (PirA) and B (PirB), which are transcribed from the pVA1 plasmid (8). The PirA/B toxins are structurally similar to insect-related binary toxins *Bacillus thuringiensis* Cry pore-forming toxin suggesting functional similarity (9). Three functional domains are responsible for the cytotoxic mechanism of the Cry toxins, and these structural features are represented in PirA/B. Cry domain I has pore-forming activity, domain II binds to receptors and domain III recognizes sugar. PirA has structural features comparative to Cry domain III, while PirB has features comparative to Cry domains I and II. Based on the similarities of PirA/B with Cry toxins, it has been suggested that the likely mechanism of pore formation caused by PirA and PirB may occur in the following sequence: 1) PirA and PirB form a complex, 2) the complex binds to sugar/receptor on a cell surface and 3) the PirA/B complex undergoes a conformational change enabling pore formation (9).

Several receptors have been identified as targets for PirA/B. In the *L. vannamei* hepatopancreatic epithelial cells, PirB has been shown to interact with glycoproteins beta-hexosaminidases subunit beta and mucin-like 5AC (10). In hemocytes of *L. vannamei*, the PirA/B toxin was able to bind to amino-peptidase N (APN) proteins. Alpha amylase-like protein may also be a PirB receptor (11). PirB also interacts with a fatty acid binding protein (Fabp), and knockdown of this gene reduced *L. vannamei* mortality, histopathological signs of AHPND, and number of VP_AHPND_ (12). However, these interactions need to be investigated further.

Research investigating the pathogenicity of VP_AHPND_ study both the host response to the bacteria and to the recombinant toxins, either individually or together. Transcriptomic studies have identified that both immune-related genes, such as pattern recognition receptor (PRRs) and hemocyanin, and a broad range of metabolic pathways, including carbohydrate metabolism, lipid metabolism and glucose metabolism, are altered in the hepatopancreas (13–17). Interestingly, there is some disagreement in the direction of expression change for some genes, such as the PRR beta-1,3-glucan-binding protein (*Bgbp*) (15, 17). Studies comparing resistant and susceptible *L. vannamei* families found that resistant shrimp typically express immune-related genes, while susceptible shrimp express genes enriched in metabolic pathways (15, 18). Therefore, changes in metabolism of the hosts cell may represent a pathogenic mechanism of the bacteria.

Omics analyses of heterogenous tissues provide an overview of expression of a tissue whereas single-cell technology allows researchers to examine the transcriptomes of individual cells. Accordingly, we set out to generate a single-cell atlas for the *L. vannamei* hepatopancreas and investigate the effects of VP_AHPND_ on the hepatopancreas at the individual cell level.

## 2 Materials and methods

### 2.1 Experimental design

To produce the hepatopancreatic cell atlas, shrimp (average weight = 13 g) were housed in 50 L tanks (23°C, 16.5 ppt salinity). Prior to sampling, the shrimp were not fed for 18 h.

For the infection response study, 70 shrimp (average weight = 19 g) were dispersed evenly between seven 50 L tanks (28°C, 4 ppt salinity). The shrimp were not fed for 24 h prior to the infection. Mock (n = 30) and infected (n = 40) treatments were randomly assigned. The mock infection shrimp were sham inoculated via reverse gavage with 100 μL tryptic soy broth containing 2% sodium chloride (TSBS), while the shrimp in the treatment group were infected with 100 μL of *V. parahaemolyticus* in TSBS. Hepatopancreatic samples were collected one hour post infection (hpi).

### 2.2 *V. parahaemolyticus* origin and culture

The D4 VP_AHPND_ strain, originating from Mexico, was used for the infection protocol (19). Briefly, the isolate was resuscitated from -80°C glycerol stock and grown for 24 h at 30°C while shaking at 175 rpm in TSBS. The 24 hr bacterial culture was then used to inoculate fresh TSBS and incubated for 6 h at 30°C with shaking at 175 rpm. The optical density (OD) at 600nm of the culture was measured, and plate counts were prepared on CHROMagar™ *Vibrio* media (CHROMagar, La Plaine Sint-Denis, France). Plate counts yielded 6.90 x 10^8^ CFU/mL.

A pilot study was conducted to select a suitable bacteria concentration for single-cell analysis based upon survival data (**Supplementary Figure 1**). Based on this pilot study, 6.90 x 10^5^ CFU/shrimp was chosen for subsequent challenge. Daily, 20% of the deceased shrimp hepatopancreas were sampled on the CHROMagar™ Vibrio plates to determine presence or absence of *V. parahaemolyticus* infection.

### 2.3 Sample preparation

The hepatopancreas was extracted from the shrimp using sterile dissecting instruments, the adventitia removed, and then gently chopped into pieces and placed into 2 mL of PluriSTEM Dispase-II solution (MilliporeSigma, St. Louis, MO) for 15 min at 37 °C. After treatment, 10 mL of phosphate buffered saline (PBS) was added, and the tissue was gently passed first through a 70 μM and then 40 μM cell sieve. The cells were centrifuged at 400 x g for 4 min at room temperature, the supernatant removed, and resuspended in 10 mL of PBS. The cells were washed two more times and counted with a TC20 automated cell counter (BioRad, Hercules, CA). Viability was assessed using an Accuri C6 Plus flow cytometer (Becton Dickinson, Franklin Lakes, NJ) with propidium iodide (PI) to ensure there was sufficient concentration and viability for library construction. The samples were diluted to the target concentration for library preparation in accordance with the 10x Genomics GEM-X Single Cell 3’ (v4) protocol (10x Genomics, Pleasanton, CA).

### 2.4 Single-cell library construction

The single-cell RNA-seq libraries were prepared using the Chromium X Instrument (10x Genomics) and the GEM-X Single Cell 3’ GEM Kit v4 (10x Genomics) following the manufacturer’s protocol. Briefly, the cells were added to the master mix and loaded into the GEM-X microfluidics chip, along with the barcoded gel beads and partitioning oil. The chip was placed into the Chromium X Instrument to generate gel beads-in-emulsion (GEMs) and immediately incubated to produce barcoded full-length cDNA. The left-over reagents were removed from the cDNA using Dynabeads MyOne SILANE (ThermoFisher Scientific, Waltham, MA) and then the cDNA was amplified via PCR. cDNA was fragmented and Illumina indexes and adapters were added via end repair, A-tailing, adaptor ligation, and then amplified via PCR. Sample cleanup and size selection of cDNA amplicons were carried out with SPRIselect (Beckman Coulter, Brea, CA). The sample quality and quantity were assessed using an Agilent TapeStation (Santa Clara, CA) with the High Sensitivity D5000 ScreenTape, and Qubit 4 Fluorometer (ThermoFisher Scientific) with the Qubit 1X dsDNA HS Assay Kit. cDNA samples were multiplexed and sequenced using the Illumina NovaSeq X+ sequencer (San Diego, CA) via a service provider (GENEWIZ/Azenta Life Sciences, South Plainfield, NJ).

### 2.5 Alignment and processing

The *L. vannamei* genome was downloaded from the NCBI (Accession #ASM4276789v1). The genome was converted to a Cell Ranger compatible format using ‘cellranger mkref’ (Cell Ranger v8.0.1). Then, scRNAseq reads were trimmed, aligned to the reference assembly, filtered, and counted to generate feature-barcode matrices using ‘cellranger count’ with intronic counts included and ‘force-cells’ set to 10,000.

Further filtering was conducted using Seurat (v5.3.0) in R (v4.5.0). First, the barcodes predicted to be duplets and multiplets using the ‘scDblFinder’ function from the scDblFinder package (v1.22.0) were removed. Then, barcodes with < 4,000 features, > 500 UMI and < 45% mitochondrial gene expression were removed. The data were normalized and regularized using the ‘SCTransform’ function (sctransform, v0.4.2) and the ‘vars.to.regress’ argument was used to reduce the contribution of the percentage of mitochondrial DNA in the principal component analysis (PCA). PCA was conducted using ‘RunPCA’.

### 2.6 Cluster analysis, cell annotation and differential gene expression analysis

The atlas samples were integrated using Seurat. First, the most variable features were identified using ‘SelectIntegrationFeatures’ then the data were prepared for integration with ‘PrepSCTIntegration’. Anchors, which are paired cells present in each dataset, were identified using ‘FindIntegrationAnchors’ and the anchors were used to perform integration with ‘IntegrateData’. The cluster analysis used dimensions 1:30 and the resolution set to 0.2. Integration for the infection study were integrated using harmony (v1.2.3) to account for batch effects, where atlas samples were in batch 1 and mock/infection samples were in batch 2. After integration, cluster analysis was carried out using 1:20 dimensions and resolution set to 0.1.

The differentially expressed (DE) genes for each cluster against all other cells in the analysis were found using the ‘FindAllMarkers’ function and Wilcoxon Rank Sum test. The P-value was adjusted for false-discovery rate (FDR), and the list was filtered for genes with an adjusted P-value < 0.05 and a log2 fold-change (log2FC) > 0.25. The list was further filtered to identify genes that were expressed in at least 50% of the cells in each cluster.

### 2.7 Pathway analysis

Over-representation analysis (ORA) was conducted using clusterProfiler (version 4.16.0) for Kyoto Encyclopedia of Genes and Genomes (KEGG) and Gene Ontology (GO) (23). ORA analysis that uses the hypergeometric test was carried out with the `compareCluster` function, using cluster as the group.

### 2.8 Treatment comparison

The cells were labeled by cluster and treatment group, then the cells in the same cluster were compared using the ‘FindMarkers’ function and Wilcoxon Rank Sum test. The resulting genes lists were filtered for genes with an adjusted P-value < 0.05 and average log2FC > 0.5, and expression in at least 25% of cells. The genes were used for pathway analyses, and the intersecting genes and pathways were visualized using UpSetR v1.4.0 (20).

## 3 Results

### 3.1 Hepatopancreas atlas

**Figure 1:**
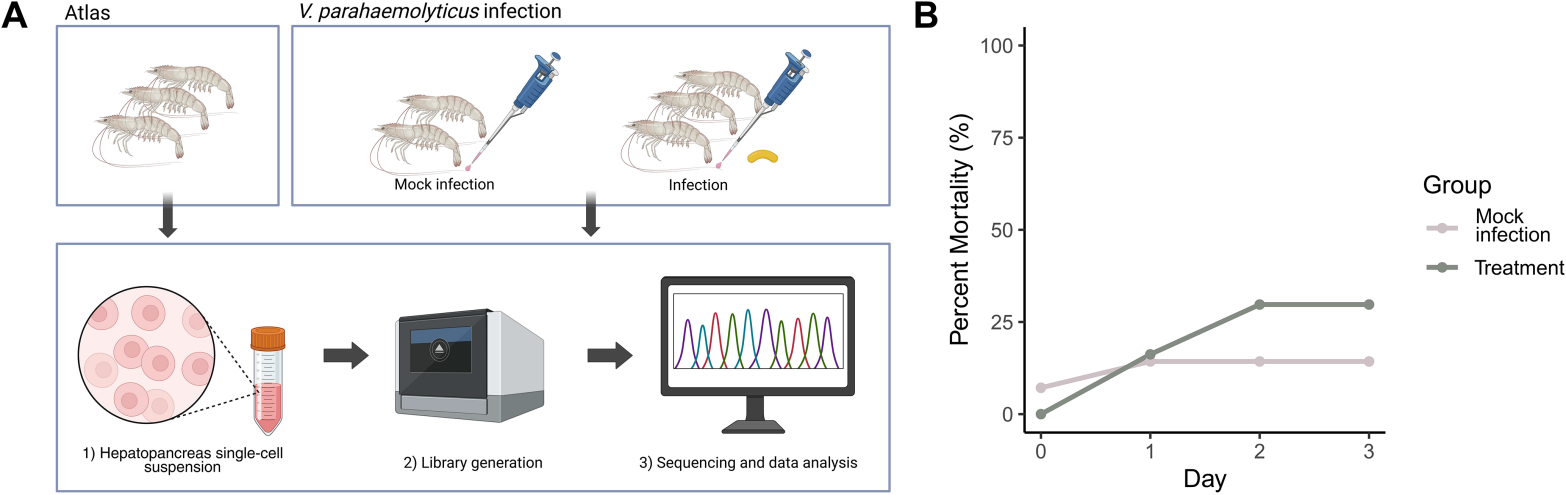
Methods used to generate the hepatopancreas single-cell transcriptomic atlas and to investigate the effects of AHPND-causing *V. parahaemolyticus*. The atlas was generated from the hepatopancreas from three shrimp, whereas for the infection study, the hepatopancreas was collected from three infected shrimp and two mock treated shrimp **(A)**. The total mortality for the infected group was 30.0% compared to 14.3% in the mock treated group **(B)**. Panel **(A)** was created in BioRender. Aldersey, J. (2026) https://BioRender.com/1zuz0tl.

**Figure 2:**
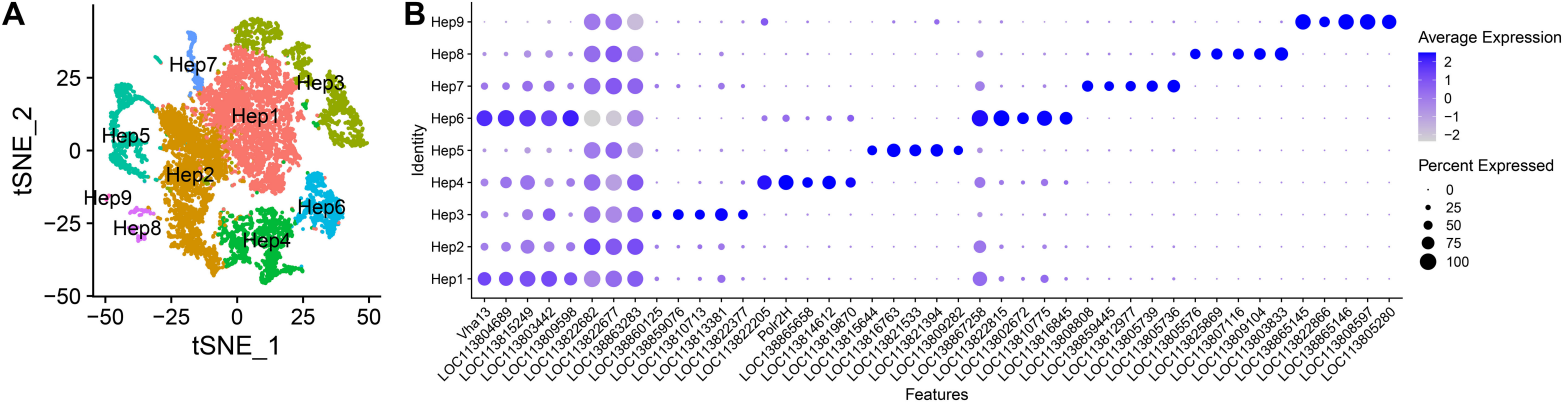
Single-cell atlas of *L. vannamei* hepatopancreatic cells (n = 3). **(A)** The nine clusters are presented with a tSNE plot. **(B)** Dot plot of the top five differentially expressed (DE) genes (top three DE genes for Hep2) for each cluster. The color represents the average expression, while dot size represents the percentage of cells expressing a gene.

The hepatopancreas from three healthy shrimp were collected to produce a single-cell transcriptomic atlas (**Figure 1A**). The libraries were sequenced to a depth of ~ 41,961 reads/cell (**Supplementary Table 1**). The libraries generated transcriptomes for 11,006 quality cells and the cluster analysis was carried out with 30 principal component dimensions and resolution of 0.2. The cluster analysis grouped the cells into nine clusters (**Figure 2A**; Hep1-9); **Supplementary Figures 2**, **3**). For each cluster, differentially expressed genes (**Supplementary Table 2**), and significant GO and KEGG pathways (**Supplementary Tables 3**, **4**) were identified.

Hep1 and Hep2 express the hemolymph proteins hemocyanin subunit (*LOC113830073*) and hemocyanin subunit-like (*LOC113823617*) (**Figure 2A**, **Supplementary Table 2**). The DE genes enriched the GO pathways glutathione transferase activity (GO:0004364), proton transmembrane transporter activity (GO:0015078), and translation (GO:0006412) (**Supplementary Table 3**). This suggests the cells are involved in detoxification of organic xenobiotics, nutrient absorption, and actively generating protein. Further, the KEGG pathway oxidative phosphorylation (pvm00190) is enriched, suggesting that the cells are involved in energy production. Hep2, despite being a distinct cluster, only has three significant DE genes which encode two ribosomal subunits (*LOC113822682*, *LOC113822677*), and NADH-ubiquinone oxidoreductase chain 1-like (*LOC138863283*) (**Figure 2A**, **Supplementary Table 2**). The cells express hemocyanin (*LOC113830073*, *LOC113823617*) and the detoxification related genes, albeit at lesser levels than Hep1.

Hep3 cells have the highest expression of heme-binding protein 1-like (*LOC138860125*), two *Bgbp* (*LOC113810713*, *LOC113813381*), hemocyanin (*LOC113830073*, *LOC113823617*) and some cells express esterase E4 (*LOC113814399*) (**Figure 2A**, **Supplementary Table 2**). Enriched pathways for Hep3 include ribonucleoprotein complex (GO:1990904), oxidative phosphorylation (GO:0006119) and lipid storage and transport (GO:0010876, GO:0006869) (**Supplementary Table 3**).

Hep4 cells expressed genes involved in cell cycle. Headcase (*heca*) and half pint (*hpf*) were highly expressed and are involved in regulating the cell cycles in Drosophila (21, 22). In Drosophila, heca is involved in controlling cell cycle progression in response to nutrient restriction (23). Splicing factors, serine/arginine repetitive matrix protein 2 (*LOC113817895*) and *hpf* suggest that there is increased mRNA processing in these cells. In support of this notion, DNA-directed RNA polymerases I, II, and III subunit Rpb8 (*Polr2H*) is also highly expressed by this cluster (**Figure 2A**, **Supplementary Table 2**). Enriched GO pathways include endosome membrane (GO:0010008), cytoplasmic vesicle membrane (GO:0030659) (**Supplementary Table 3**) while the KEGG pathways include glycosaminoglycan degradation (pvm00531) and lysosome (pvm04142) (**Supplementary Table 4**).

Cells in Hep5 expressed immune related genes, such as prophenoloxidase (proPO; *LOC138859383*), anti-lipopolysaccharide factor (ALF; *LOC113800363*, *LOC113820510*, *LOC113810108*, *LOC113810045*), C-type lectin (*LOC113823075*, *LOC113812219*) and lysozyme C (*LOC113802295*), typical of hemocytes (**Supplementary Table 2**). ProPOs are expressed by mature effector cells. The enriched pathways further suggest the cells have immune functions. The enriched KEGG pathways include bacterial invasion of epithelial cells (pvm05100), hormone signaling (pvm04081), efferocytosis (pvm04148), phagosome (pvm04145) and Toll-like receptor signaling pathway (pvm04620) (**Supplementary Table 4**).

The genes highly expressed by Hep6 cells had enriched GO pathways of inorganic cation transmembrane transporter activity (GO:0022890), cytoskeleton organization (GO:0007010) and vacuole (GO:0005773) (**Supplementary Table 3**), and KEGG pathways oxidative phosphorylation (pvm00190), phagosome (pvm04145), lysosome (pvm04142) and metabolism of xenobiotics by cytochrome P450 (pvm00980). The cells highly express genes encoding 10 V-type proton ATPase subunits which are involved in acidification of vacuoles, and digestive enzymes astacin (*LOC113802672*), esterase (*LOC113818979*) and trypsin (*LOC113822075*) (**Figure 2**) (**Figure 2A**, **Supplementary Table 2**). Furthermore, cells expressed endocytosis related genes *Rab11*, *Rab5*, *Rab1*. Overall, the gene expression and pathway analysis suggest that these cells internalize substances from the external environment and digest this material.

Hep7 highly expressed digestive enzymes astacin (*LOC113826331*, *LOC113826330*, *LOC113826343*, *LOC113819445*), procathepsin L (*LOC113808797*, *LOC113808808*), alpha-amylase (*LOC113817723*), trypsin (*LOC113815565*, *LOC113815556*, *LOC113825851*), chymotrypsins (*LOC138859445*, *LOC113805739*, *LOC113805736*) and chitinase (*LOC113817260*, *LOC113817261*, *LOC113817258*) (**Figure 2A**, **Supplementary Table 2**). Furthermore, this cluster highly expresses C-type lectins (CTLs), C-type lectin domain family 7 member A (*LOC113812977; Clec7a*), alpha-N-acetylgalactosamine-specific lectin-like (GalNAc-specific lectin; *LOC138862967*) and galactose-specific lectin nattectin-like (Nattectin-like; *LOC138864390*). The enriched GO pathways included macromolecule catabolic process (GO:0009057) and endopeptidase activity (GO:0004175) (**Supplementary Table 3**) which emphasizes the cells function in digestion.

Hep8 highly expresses genes that encode structural proteins of muscle including paramyosin (*LOC113809104*), connectin/titin (*LOC113812009*, *LOC113807395*), Z band alternatively spliced PDZ-motif protein 52 (*Zasp52*), muscle-specific protein 300 kDa (*LOC113823000*), tropomyosin (*LOC113820940*) and myosin heavy chain 10 (*zip*) (**Supplementary Table 2**). The most significant KEGG pathway is cytoskeleton in muscle cells (pvm04820) (**Supplementary Table 4**).

The cells in Hep9 express genes related to synapse function. These include uncharacterized protein (*LOC138865145*), gamma-aminobutyric acid (GABA) type B receptor subunit 2-like (*LOC113822866*), sidekick cell adhesion molecule (*sdk*) (24), regulator of G-protein signaling 7-like (*LOC113828644*) (25) and syntaxin-binding protein tomosyn (*Tomosyn*) (**Figure 2A**, **Supplementary Table 2**). The protein transcribed by *LOC138865145* (XP_069990618.1) contains a “ligand-binding domain of GABAb receptors”, therefore, this gene may transcribe another GABA receptor subunit. GABA receptors are present in pre- and post-synaptic sites in the mammalian central nervous system. Interestingly, the cells also expressed RAS oncogene family member Rab3 (*rab3b*), which are highly expressed by human enteroendocrine cells, pancreatic endocrine cells and inhibitory neurons (26, 27). Genes in the *rab3* family were shown to regulate exocytosis in mouse cell lines (28). Innexin, a gap junction protein which enables communication between adjacent cells, is also highly expressed.

### 3.2 VP_AHPND_ infection analysis

The hepatopancreas from three infected shrimp and two control shrimp were collected to carry out single-cell transcriptomic analysis (**Figure 1A**). The total mortality for the infected group was 30.0% compared to 14.3% in the mock treated group (**Figure 1B**). Swabs collected from the hepatopancreas of deceased shrimp confirmed that the shrimp in the infected group were positive for *V. parahaemolyticus*, while the control shrimp were negative.

**Figure 3:**
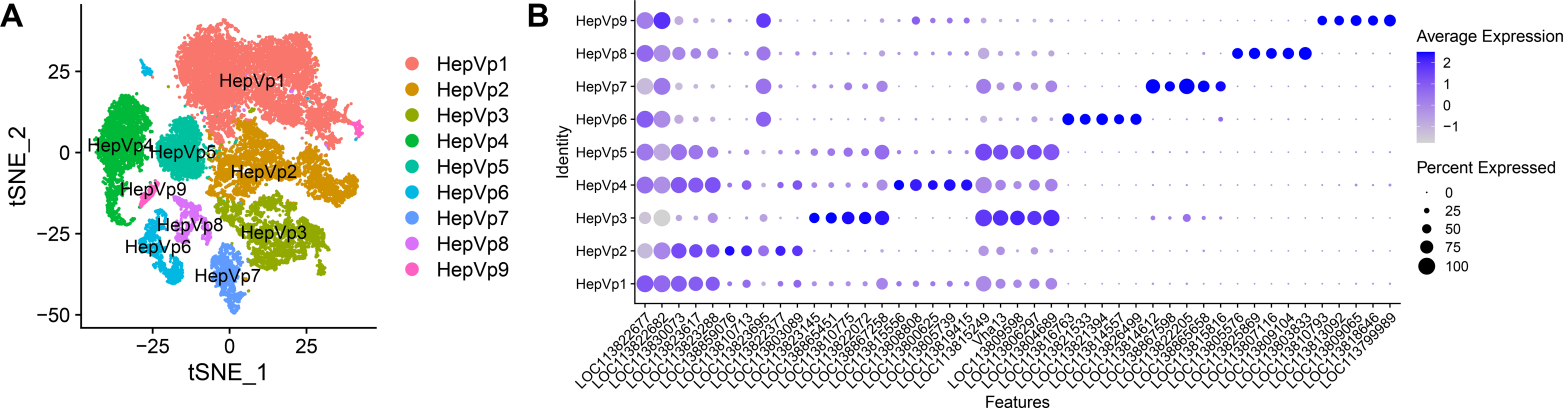
Cluster analysis of hepatopancreas cells from infected (n = 3) and control (n = 5) shrimp. **(A)** nine clusters are presented with a tSNE plot. **(B)** Dot plot of the top five differentially expressed (DE) genes for each cluster. The color represents the average expression, while dot size represents the percentage of cells expressing a gene.

The libraries from the infection study, created from hepatopancreas cells from three VP_AHPND_ infected shrimp and two mock treated shrimp, were sequenced to a depth of ~66,018 reads/cell (**Supplementary Table 1**). These five libraries generated transcriptomes for 16,368 quality cells were integrated with the atlas samples taking batch effects into consideration, for a total of 27,374 cells (**Supplementary Figures 4**, **5**). Nine clusters were identified in the VP_AHPND_ infection study (**Figure 3**). Overall, the same populations were present between the atlas and infection study datasets (**Supplementary Figure 6**, **Supplementary Tables 5**–**7**).

**Figure 4:**
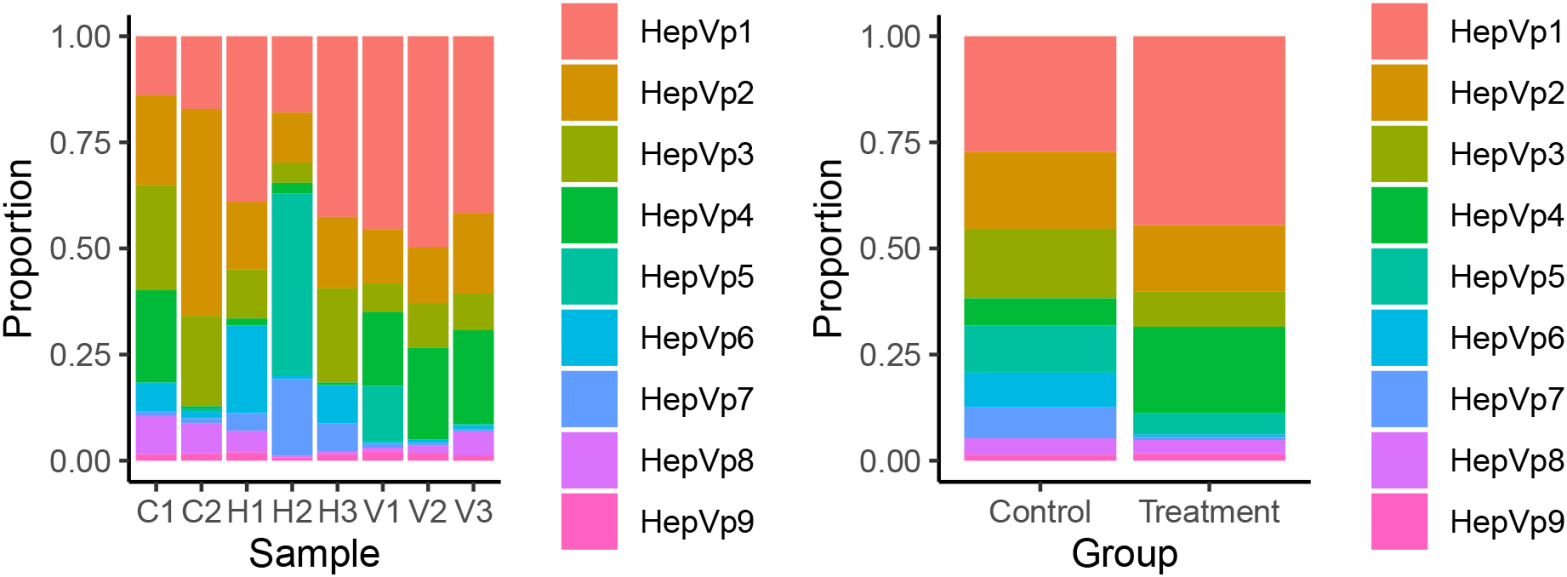
Sample and treatment cell compositions. **(A)** The cell composition by sample. The X-axis indicates the sample while the Y-axis indicates the proportion. H1–3 are the atlas samples, C1–2 are the mock treatment samples and V1–3 are they infected samples. **(B)** The cell composition by treatment. The control group includes the atlas and mock treatment samples (n = 5) while the treatment group were infected with the *V. parahaemolyticus* (n =3).

**Figure 5:**
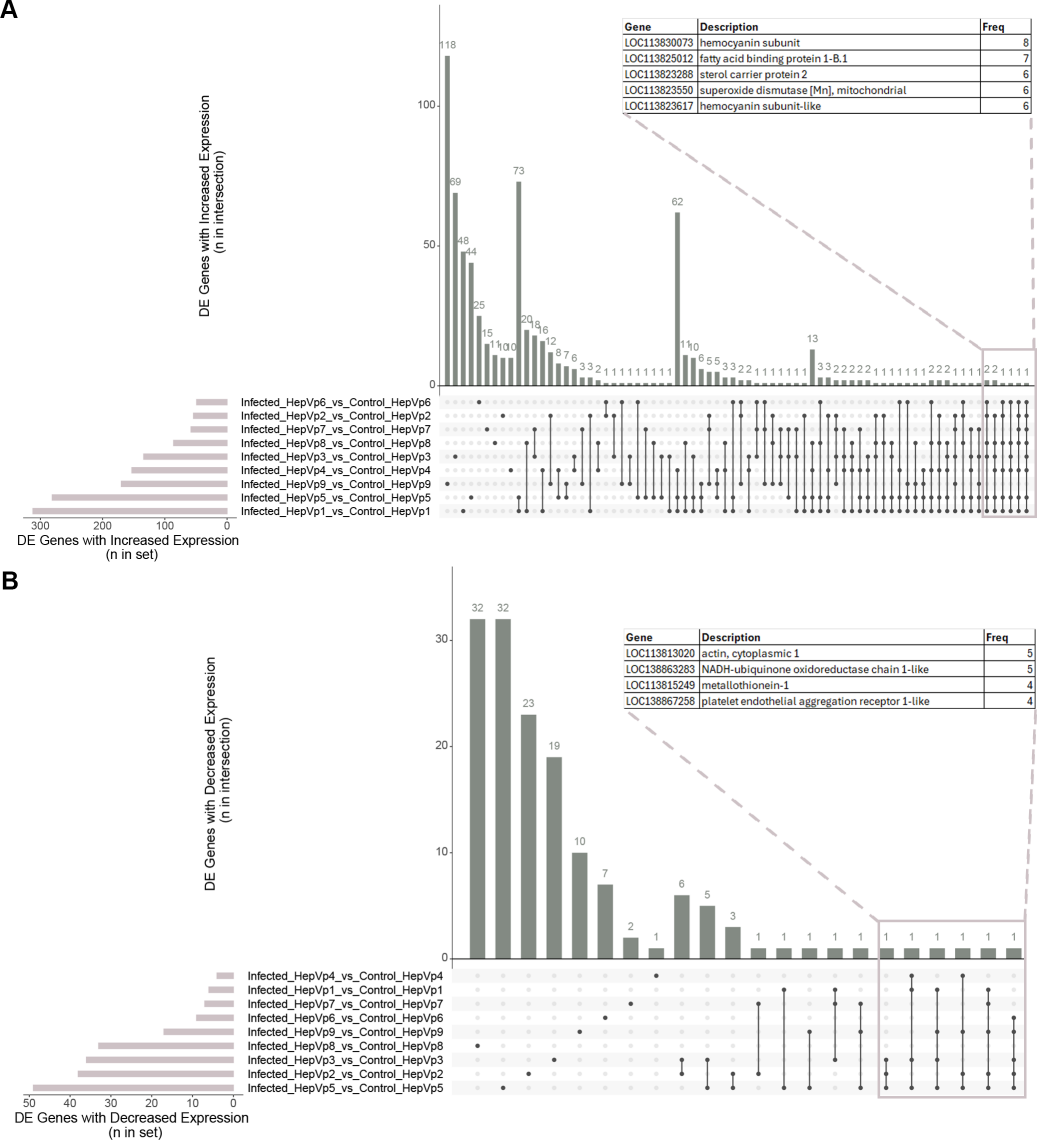
UpSet plot visualizing common (‘intersecting’) differentially expressed genes for each cluster comparison between the infected and control group. Intersecting differentially expressed genes with increased expression **(A)** and decreased expression **(B)** are displayed in the column graph with intersection size on the y-axis. The intersections are indicated by the matrix below the x-axis, where the rows represent the sets and the columns represent the intersection. Black dots indicate that a set is included in the intersection whereas grey circles indicate the set is not included. The size of the intersect (number of genes) is indicated by the columns and number above the column. The total number of differentially expressed genes for each cluster comparison is shown in the bar graph. Selected genes are displayed in the table and “Freq” indicates the number of gene sets in which the gene is included. The full list of gene frequencies is presented in **Supplementary Table 9**.

**Figure 6:**
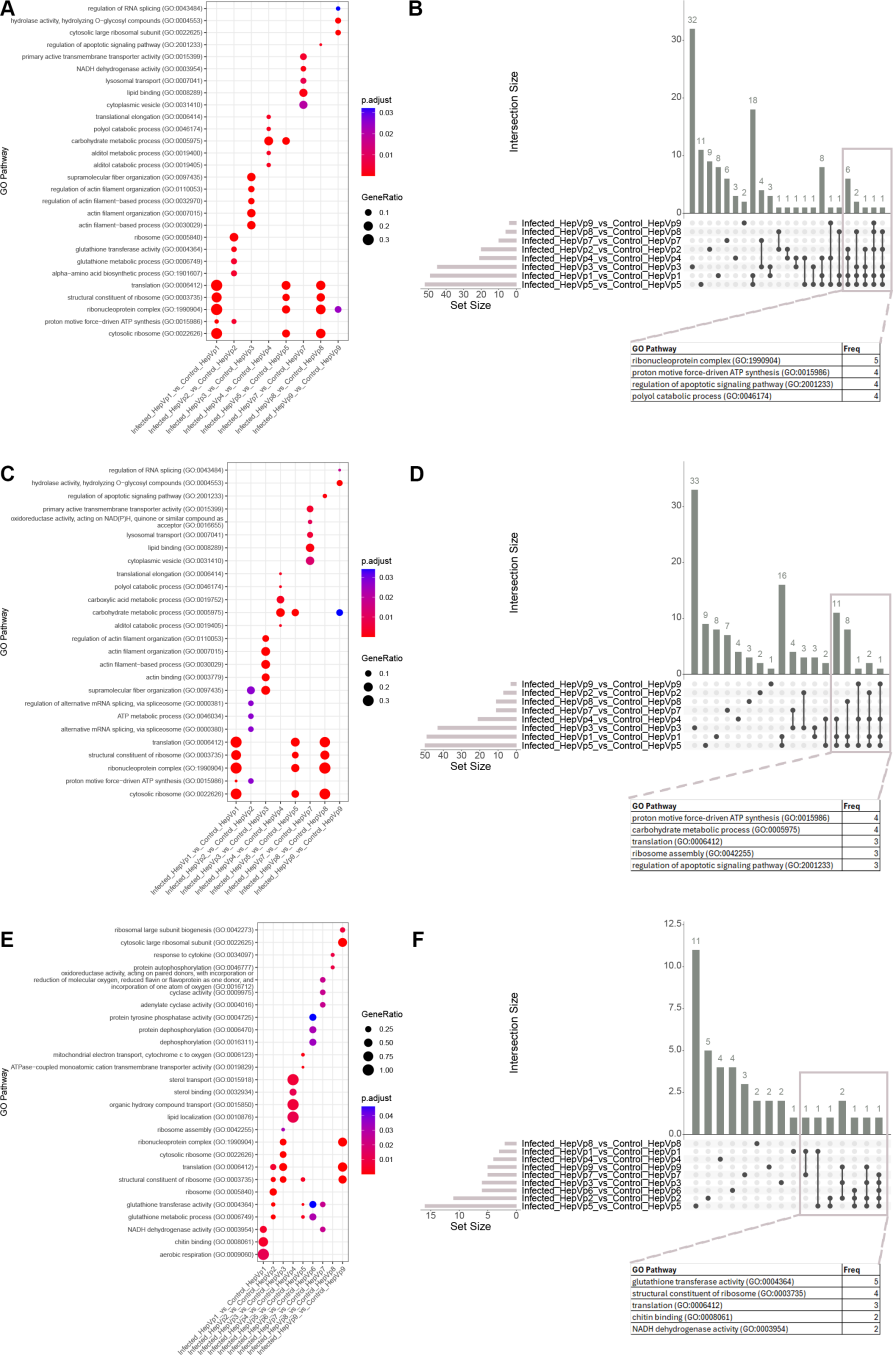
GO enrichment from overrepresentation analysis. **(A, C, E)** Dot plots displaying the top five significantly enriched GO pathways for each comparison using all differentially expressed (DE) genes **(A)**, DE genes with increased expression **(B)**, and DE genes with decreased expression in infected cells **(C)** as input. **(B, D, F)** UpSet plot visualizing common (‘intersecting’) enriched GO pathways for each cluster comparison between the infected and control group. Intersecting pathways for all differentially expressed genes **(B)**, genes with significantly increased expression **(D)** and genes with significantly decreased expression **(F)** are displayed in the column graph with Intersection Size on the y-axis. The intersections are indicated by the matrix below the x-axis, where the rows represent the sets and the columns represent the intersection. Black dots indicate that a set is included in the intersection whereas grey circles indicate the set is not included. The size of the intersect (number of pathways) is indicated by the columns and number above the column. The total number of significant pathways for each cluster comparison is shown in the bar graph. Selected genes are displayed in the table and “Freq” indicates the number of gene sets in which the gene is included. The full list of GO pathway frequencies is presented in **Supplementary Table 12**.

**Figure 7:**
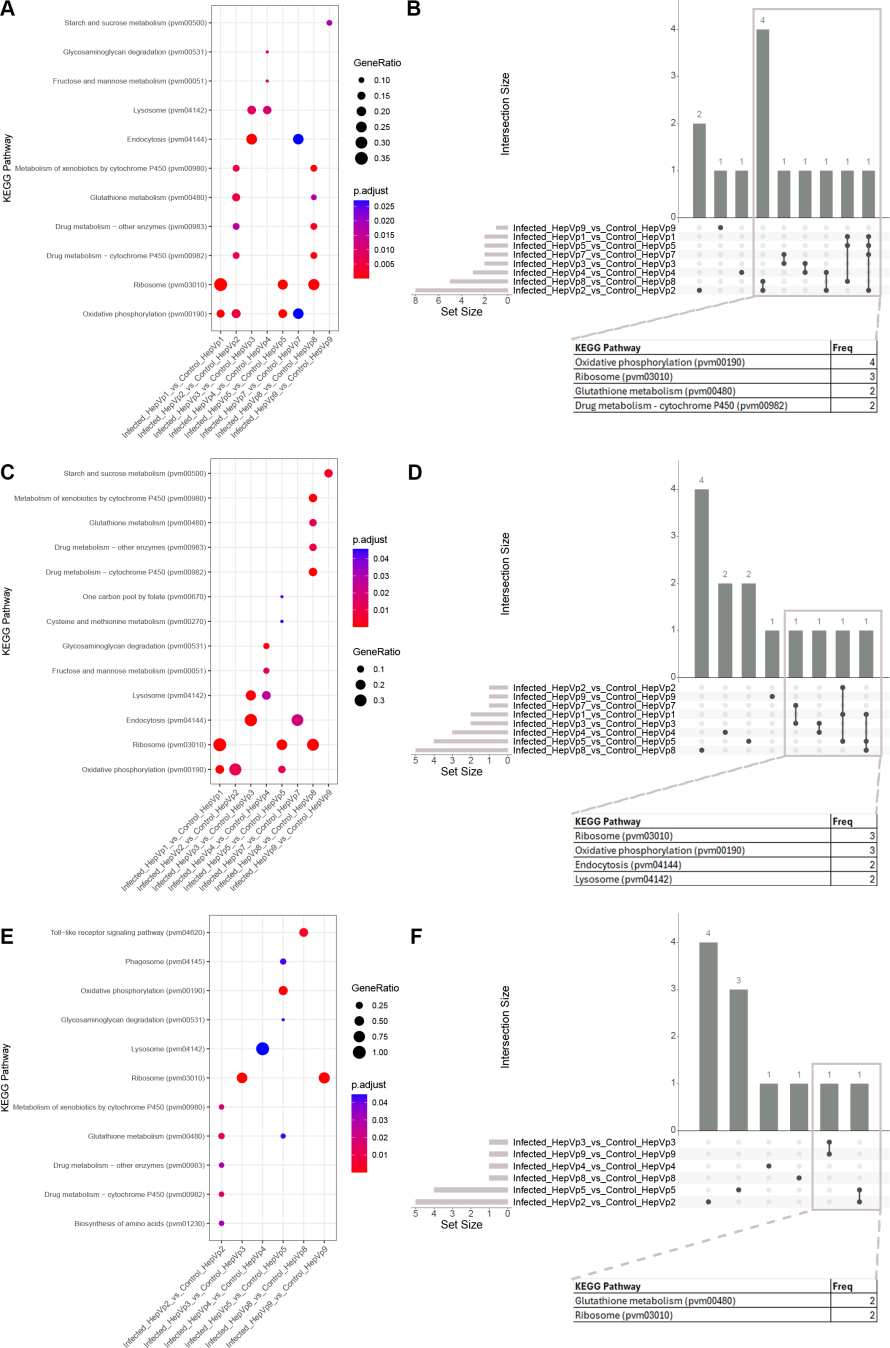
KEGG enrichment from overrepresentation analysis. **(A, C, E)** Dot plots displaying the top five significantly enriched KEGG pathways for each comparison using all differentially expressed (DE) genes **(A)**, DE genes with increased expression **(B)**, and DE genes with decreased expression in infected cells **(C)** as input. **(B, D, F)** UpSet plot visualizing common (‘intersecting’) enriched GO pathways for each cluster comparison between the infected and control group. Intersecting pathways for all differentially expressed genes **(B)**, genes with significantly increased expression **(D)** and genes with significantly decreased expression **(F)** are displayed in the column graph with Intersection Size on the y-axis. The intersections are indicated by the matrix below the x-axis, where the rows represent the sets and the columns represent the intersection. Black dots indicate that a set is included in the intersection whereas grey circles indicate the set is not included. The size of the intersect (number of pathways) is indicated by the columns and number above the column. The total number of significant pathways for each cluster comparison is shown in the bar graph. Selected genes are displayed in the table and “Freq” indicates the number of gene sets in which the gene is included. The full list of KEGG pathway frequencies is presented in **Supplementary Table 13**.

The effect of *V. parahaemolyticus* was assessed by comparing the cellular composition and gene expression of the hepatopancreas between infected shrimp (n = 3) and control shrimp (n = 5) (**Figures 4**, **5**, **Supplementary Table 8**). Comparisons of cell type proportions show that the infected shrimp had higher proportions of HepVp1 (Control = 27.3%; Infected = 44.5%) and HepVp4 (Control = 6.4%; Infected = 20.3%), lower proportions of HepVp3 (Control = 16.4%; Infected = 8.3%), HepVp5 (Control = 11.1%; Infected = 5.1%), HepVp6 (Control = 8.1%; Infected = 0.6%) and HepVp7 (Control = 7.4%; Infected = 0.7%) (**Figures 4**), and approximately equal proportions of HepVp2 (Control = 18.1%; Infected = 15.7%), HepVp8 (Control = 3.8%; Infected = 3.1%) and HepVp9 (Control = 1.4%; Infected = 1.7%). The differentially expressed genes were used as input for over-representation analysis of GO (**Figure 6**, **Supplementary Table 10**) and KEGG pathways (**Figure 7**, **Supplementary Table 11**).

To investigate overarching patterns, genes and pathways significant across multiple clusters were identified. UpSet plots (20) were generated to visualize the number of common (‘intersecting’) significant genes and pathways for the infected versus control comparisons carried out by cluster (**Figures 5**–**7**). The number of times a gene (**Supplementary Table 9**) and pathway (**Supplementary Tables 12**, **13**) is significant is also presented as supplementary data.

The UpSet plot of DE genes showed that there are some genes that are expressed across many clusters (**Figure 5**, **Supplementary Tables 8**, **9**). Hemocyanin (*LOC113830073*; HepVp1-8) was DE for eight clusters, and hemocyanin subunit-like (*LOC113823617*; HepVp1-2,4-6,8) was DE for six clusters (**Figure 5A**, **Supplementary Table 9**). Four genes involved in acyl CoA transport and metabolism, fatty acid binding protein 1-B.1 (*Fabp*; *LOC113825012*; seven clusters), sterol carrier protein 2 (*LOC113823288;* six clusters), acyl-CoA Delta-9 desaturase (*LOC113822166*; four clusters) and acyl-CoA binding protein 1 (*Acbp1*; four clusters) are highly expressed in the infected group compared to the control group (**Supplementary Table 9**). Superoxide dismutase [Mn], mitochondrial (*LOC113823550*) and aldehyde dehydrogenase type III (*Aldh-III*) has increased expression in infected cells from six and five comparisons, respectively, indicating the cells are reacting to oxidative stress (29, 30) (**Supplementary Table 9**). Genes that were downregulated across multiple clusters included actin, cytoplasmic 1 (*LOC113813020*) and NADH-ubiquinone oxidoreductase chain 1-like (*LOC138863283*), metallothionein-1 (*LOC113815249*), platelet endothelial aggregation receptor 1-like (*LOC138867258*; *Pear1*) and an uncharacterized protein (*LOC138867259*) (**Figure 5B**, **Supplementary Table 9**). *Pear1* and *LOC138867259* are involved in chitin binding.

For the GO and KEGG over-representation analyses, three input gene sets were used: 1) all DE genes, 2) genes with increased expression, and 3) genes with decreased expression. Then, UpSet plots were generated for each of these analyses (**Figures 6**, **7**), which showed that some clusters have shared responses to infection.

When all genes were analyzed, GO pathways involved in ribosome structure (GO:1990904, GO:0003735), ATP metabolism (GO:0006754, GO:0046034), apoptosis (GO:2001233), alditol/polyol catabolism (GO:0046174, GO:0019400) were significant for four to five cluster comparisons (**Figure 6B**, **Supplementary Table 12**). For KEGG pathways, four comparisons had enriched the pathway oxidative phosphorylation (pvm00190), and three comparisons enriched ribosome (pvm03010) (**Figure 7B**, **Supplementary Table 13**). When only the genes with greater expression were considered, the GO pathways relating to ATP metabolism and carbohydrate metabolism were significant for four comparisons, and pathways relating to translation, apoptosis, metabolism (alditol, GO:0019405; polyol, GO:0046174; carboxylic acid, GO:0019752, GO:0046394; monosaccharide, GO:0046364) were significant for three comparisons (**Figure 6D**, **Supplementary Table 12**) and the KEGG pathways oxidative phosphorylation (pvm00190) and ribosome (pvm03010) were significant for three comparisons (**Figure 7D**, **Supplementary Table 13**). When only the genes with lower expression were considered, GO pathways related to glutathione activity (GO:0004364; GO:0006749) and ribosomes (GO:0003735; GO:1990904) were shared between three to five comparisons (**Figure 6F**, **Supplementary Table 12**), and the KEGG pathway glutathione metabolism (pvm00480) and ribosome (pvm03010) were shared between three comparisons (**Figure 7F**, **Supplementary Table 13**).

Next, we explored the unique cluster specific responses to infection. Infected_HepVp1 expressed genes involved in immune response that may interact with PirA/B toxins. Among the top DE genes for Infected_HepVp1 compared to Control_HepVp1 are PRRs, including hepatic lectin (*LOC113805524*), CTLs (*Clec6A; LOC113812976*, *Clec7A*, *Clec4F; LOC113805525, Clec17A-like; LOC138866223*, Nattectin-like), macrophage mannose receptor 1 (*Mrc1*; *LOC113820644*) and *Bgbp* (*LOC113807222*) (**Supplementary Table 8**). The Infected_HepVp1 cells also highly expressed trypsin (*LOC113825851*, *LOC113815556*), chymotrypsin (*LOC138859443*, *LOC113805739*, *LOC113805736*), astacin (*LOC113826343*, *LOC113826331*), carboxypeptidase B (*LOC113802097*), lysozyme (*LOC113805933*), procathepsin L (*LOC113808797*, *LOC113808808*), and legumain (*LOC113827866*, *LOC113827868*) compared to the control (**Supplementary Table 8**). Another gene encoding protein that may also interact with the PirA/B toxin includes beta-hexosaminidase subunit alpha (*LOC113811731*). Chitin binding (GO:0008061) was enriched by upregulated genes for Infected_HepVp1 and included four peritrophins, three chitinase-3-like protein 1 (*Chi3l1*) and four proteins with chitin-binding domains (**Supplementary Table 10**).

The top DE gene for Infected_HepVp2 cells was sorbitol dehydrogenase (*LOC113814344*) an enzyme involved in converting sorbitol to fructose (**Supplementary Table 8**). Additionally, several transcription factors are highly expressed, including clockwork orange (*LOC113811900*; *cwo*), homeobox protein dve-1 (*LOC113804003*), nuclear factor of activated T-cells 5 (*NFAT*), myocyte enhancer factor 2 (*Mef2*), microphthalmia-associated transcription factor (*Mitf*) and transcriptional coregulator yorkie (*yki*) (**Supplementary Table 8**). Genes with greater expression for Infected_HepVp2 enriched GO pathways involved in actin organization (GO:0007015) whereas genes with lower expression enriched carbohydrate catabolic process (GO:0016052) (**Figure 6**, **Supplementary Table 10**).

HepVp3 cells are characterized by expression of killer cell lectin-like receptor 5 (*LOC138865451*), *Rab5*, *Rab1*, *Rab11* and GDP dissociation inhibitor (*Gdi*) and these were all more highly expressed by the Infected_HepVp3 cells (**Supplementary Table 5**). Infected_HepVp3 had higher expression of early endosome antigen 1 (*Eea1*; *LOC113828458*), and genes expressed by mammalian innate immune cells including ATP Binding Cassette Subfamily A Member 5 (*LOC113801903*), lymphocyte Antigen 75 (*Ly75*; *LOC113822862*), macrosialin (*Cd68*; *LOC113808348*) and soluble scavenger receptor cysteine-rich domain-containing protein (*Ssc5d*; *LOC113824628*) (**Supplementary Table 8**). Genes that were more highly expressed by Infected_HepVp3 cells enriched pathways included actin filament organization (GO:0007015), vesicle-mediated transport (GO:0016192), and lipid binding (GO:0008289) (**Figure 6**, **Supplementary Table 10**). The enriched KEGG pathways were endocytosis (pvm04144) and lysosome (pvm04142) (**Figure 7**, **Supplementary Table 11**). The GO pathways involved in metabolic processes included peptide metabolic process (GO:0006518) and very long-chain fatty acid metabolic process (GO:0000038) and purine nucleotide metabolic process (GO:0006163) (**Supplementary Table 10**).

Infected_HepVp4 cells more highly expressed genes with immune functions compared to the Control_HepVp4 cells. These genes included *Bgbp* (*LOC113807222*), lectins (*LOC113805525*, *LOC113805524*) and trypsins (*LOC113825851*, *LOC113815556*) (**Supplementary Table 8**). In addition, the infected cells also highly expressed genes encoding proteins that protect against apoptosis and stress, including lifeguard 1 (*LOC113823324*), arginine kinase (*LOC113816366*), elongation factor 1-alpha (*LOC113820946*) and crustacyanin-A2 subunit (*LOC113810219*) (**Supplementary Table 8**). The enriched GO pathways organophosphate metabolic process (GO:0019637) (**Supplementary Table 10**). Enriched KEGG pathways were lysosome (pvm04142), glycosaminoglycan degradation (pvm00531) and fructose and mannose metabolism (pvm00051) (**Figure 7**, **Supplementary Table 11**).

The cells in HepVp5 were mainly from samples H2 and V1(**Supplementary Figure 4**). The cluster is mainly characterized by increased expression of ribosomal proteins.

Cells in HepVp6 expressed hemocyte-related genes ALFs (*LOC113800363*, *LOC113820510*), CTL (*LOC113823075*) and lysozyme C (*LOC113802295*), typical of hemocytes (**Supplementary Table 8**). Genes with greater expression in Infected_HepVp6 cells were involved in immune functions and response to stress including Krueppel-like factor 13 (*LOC113799918*), activating transcription factor 3 (*LOC113808698*), low-density lipoprotein receptor (*LOC113811552*), NF-kappa-B inhibitor cactus (*LOC113823636*) and legumain-like (*LOC113827868*) (**Supplementary Table 8**). Comparing Infected_HepVp6 and Control_HepVp6 yielded few significant GO and KEGG pathways.

HepVp7 are characterized by two MAM and LDL-receptor class A domain-containing protein 2 proteins (*LOC113814612*, *LOC138867598*), GTPase-activating Rap/Ran-GAP domain-like protein 3 (*LOC113822205*), rap guanine nucleotide exchange factor-like (*LOC138865658*) and runt-related transcription factor 3 (*LOC113815816*) (**Supplementary Table 5**). When Infected_HepVp7 was compared to Control_HepVp7, the genes with increased expression enriched GO pathways relating to internalization of substance including lysosomal transport (GO:0007041), cytoplasmic vesicle (GO:0031410), lipid binding (GO:0008289), GTPase activator activity (GO:0005096) and syntaxin binding (GO:0019905) (**Figure 6**, **Supplementary Table 10**). The enriched KEGG pathway was endocytosis (pvm04144) (**Figure 7**, **Supplementary Table 11**).

The upregulated gene expression of Infected_HepVp8 enriched the enriched GO pathways included glutathione transferase activity (GO:0004364) (**Supplementary Table 10**) and KEGG pathways included drug metabolism - cytochrome P450 (pvm00982) and glutathione metabolism (pvm00480) (**Figure 7**, **Supplementary Table 11**). The top expressed genes included *Mrc1* (*LOC113820644*) and among the down regulated genes in the infected group were immune-related genes including nuclear factor NF-kappa-B p105 subunit (*Nfkb1*; *LOC113806131*) and NF-kappa-B inhibitor cactus (*LOC113823636*) (**Supplementary Table 8**). Muscle-related genes such as tropomyosin (*LOC113809272*) and paramyosin (*LOC113809104*) also had lower expression in the infected group compared to the control group.

HepVp9 was characterized by the expression of RYamide receptor (*LOC113810793*), von Willebrand factor A domain-containing protein 7 (*LOC113813092*, *LOC113806365*), vitelline membrane outer layer protein 1 (*LOC113819458*) and chondroitinase-AC (*LOC113799989*) (**Supplementary Table 5**). When Infected_HepVp9 was compared to Control_HepVp9, the genes with greater expression enriched GO pathways regulation of RNA splicing (GO:0043484), hydrolase activity, hydrolyzing O-glycosyl compounds (GO:0004553) (**Figure 6**, **Supplementary Table 10**), and the KEGG pathway starch and sucrose metabolism (pvm00500) (**Figure 7**, **Supplementary Table 11**). Control_HepVp9 expressed uncoupling protein (*Bmcp*), alkaline phosphatase (*LOC113808761*), *Mrc1* (*LOC113820644, LOC113818671*), alpha-amylases (*LOC113825898*, *LOC113817723*, *LOC113804635*, *LOC113825904*), and rho GTPase activating proteins (*RhoGAP19D*, *LOC113803245*, *LOC113821008*) (**Supplementary Table 8**).

## 4 Discussion

Pacific white shrimp are one of the most highly produced aquaculture species worldwide (1). The industry has been significantly impacted by the infectious disease AHPND caused by VP_AHPND_. VP_AHPND_ primarily targets the hepatopancreas, causing necrosis by the pore-forming PirA/B toxins. Several studies have investigated changes in gene expression of hepatopancreatic cells in response to both toxins and bacteria. However, the organ comprises several cell types and the bacteria may affect the cell types differently. The shrimp immune system lacks adaptive responses and consists of physical barriers, cellular, and humoral components (31). Hemocytes are the major immune cell in shrimp and responses include phagocytosis and apoptosis (32). Immune molecules, primarily found in the hemolymph, include antimicrobial peptides, the proPo system and proteases. The hepatopancreas is also responsible for the production of some of these hemolymph proteins (33). Our research examined the gene expression changes of hepatopancreas cells in response to VP_AHPND_ infection.

Our atlas dataset was produced from hepatopancreas cells from three healthy shrimp (*L. vannamei*), and nine clusters were found. Most clusters had distinct transcriptional profiles, illuminating the potential function of the cells which may represent cell-types or cell-states. The known cell types of the hepatopancreas are E-cells, R-cells, F-cells, B-cells and M-cells. Additionally, hemocytes, phagocytic cells and smooth muscle cells are associated with hepatopancreas. We attempted to identify hepatopancreas cells; however, current cell-type markers and functional characterization were insufficient for cell annotation in most cases, excluding hemocytes which are well characterized. Furthermore, the expression of known cell markers for a cell type were not expressed by a single cluster. For example, F-cells were demonstrated to express *Bgbp* and hemocyanin in normal conditions in *L. vannamei* via *in situ* hybridization (7) and known to secrete digestive enzymes (5, 34). However, *Bgbp* and hemocyanin were expressed by Hep3 and digestive enzymes were expressed by Hep7, indicating these may be two distinct cell types. Some cells, such as B-cells and M-cells, do not have distinct markers or clearly defined functions. Therefore, there is not enough functional knowledge regarding hepatopancreas cell gene expression to definitively annotate the cells, and further experiments visualizing expression in the cells, such as using *in situ* hybridization, are required to confirm the identity of these transcriptionally distinct clusters.

Our infection study examined the changes of cell types and cell proportions of the hepatopancreas across VP_AHPND_ infected and control shrimp one hour post infection. Our data showed there were changes in the proportion of cells between treatment groups with both increased and decreased proportions in response to infection. The clusters with lower cell numbers (HepVp3/6/7) in the infected population may be undergoing apoptosis or directly targeted by the VP_APHND_ PirA/B toxins. The cells from the infected group did not enrich apoptosis related pathways suggesting that cell death is more likely related to infection. One of these clusters, HepVp6, represent hemocytes as they express proPO, ALFs, CTL and lysozyme (35). A previous infection study found that the total hemocyte count was lower in infected shrimp compared to mock treated shrimp (36). The clusters with higher cell numbers (HepVp1/4) in the infected group may be the result of proliferation or differentiation. These are characterized by high expression of genes transcribing humoral proteins such as proteases (trypsin, chymotrypsin, cathepsin L, legumain), lysozyme and CTLs, which suggest the cells mainly have humoral immune properties. Increases in the number of cells are expected if the cells are not specifically targeted by the pathogen and are responding immunologically. For example, the count of proPO expressing hemocytes increased in red swamp crayfish (*Procambarus clarkii*) and Japanese mittin lobster (*Parribacus japonicus*) in response to lipopolysaccharide stimulation (35). Considering the hemocyte population was almost eliminated in the infected population, other immune mechanisms may be more important for defending against VP_AHPND_.

Cluster HepVp3 exhibited expression consistent with endocytosis which could represent phagocytosis or internalization of nutrients from the hepatopancreas lumen. Signatures of endocytosis such as *Ssc5d* and *Rab5*, and genes with immune functions including killer cell lectin-like receptor 5, *Ly75*, and *Cd68* were expressed. *Ssc5d* is a scavenger receptor, and these receptors that can bind multiple ligands for the removal of non-self and apoptotic cells (37, 38). Scavenger receptors in several crustacean species are shown to act as PRRs and are involved in phagocytosis (39, 40). *Rab5* is a small GTPase marker of early endocytosis and regulates vesicle trafficking; its effector molecule *Eea1* is also highly expressed (41). In response to infection, the pathways relating to endocytosis and lysosomes are enriched. Therefore, the expression of this HepVp3 indicates that it could have potential immune functions beyond endocytosis of nutrients, but further experiments are required to determine if these are phagocytic immune cells or hepatopancreatic cells (such as B-cell) with infrastructure for both nutrient and immune-related endocytosis. As these cells are responding to the infection of VP_AHPND_, this characterization is important to determine.

In response to infection, the hepatopancreatic cells increased expression of PRRs. Many PRRs were found in the dataset, and a subset had increased expression in Infected_HepVp1/4/5/6/8/9. These include *Bgbp* and a suite of CTLs (including *Mrc1*, *Clec4F*, hepatic lectin, GalNAc-specific lectin, Nattectin-like). PRRs are ubiquitously expressed by cells and initiate an innate immune response upon activation. Activated pathways depend on the pathogen and include the Toll pathway, immune deficiency (IMD) pathway and JAK/STAT pathway (31). Hepatic lectin was shown to function as a PRR for both Gram-negative and Gram-positive bacteria in *Danio rerio* (42). The *Mrc1* is involved in pattern recognition but also has functions in other processes such as homeostasis (43). While the role of *Clec4F* interaction in shrimp response to white spot syndrome virus (WSSV) has been recently characterized (44), its role in bacterial infections is not well understood. Nattectin-like was recently characterized in red swamp crayfish (*Procambarus clarkii*) and RNA interference caused significant downregulation of immune genes (45).

Beyond cellular immunity, the cells exhibited strong expression of humoral immunity components. The humoral immunity factors are soluble effector molecules that are secreted to combat pathogens and include proPO, lectins, agglutinins and anti-microbial peptides (AMPs). The main factors with increased expression were hemocyanin, proteinases, lysozyme and CTLs.

After infection, hemocyanin expression was increased in eight clusters. Hemocyanin, in addition to being an oxygen carrier, has immune functions. Hemocyanin can agglutinate VP_AHPND_, neutralize the toxins effect *in vivo*, and was demonstrated to specifically interact with PirA (46, 47). In response to VP_AHPND_, expression of hemocyanin rapidly increases within 3 hpi and significantly declines after 6 hpi (46). Other infection studies have demonstrated that VP_AHPND_ exposure regulates hemocyanin expression in *L. vannamei* (13, 14, 17, 48, 49). Resistant *L. vannamei* shrimp families have greater baseline expression levels of hemocyanin than shrimp from susceptible families, indicating the importance of hemocyanin in host defense against VP_AHPND_ (15). However, several studies report that hemocyanin had reduced expression in infected shrimp (14, 17, 49). There are 21 hemocyanin annotations in the reference genome used herein, and some conflicting reports may be due to the specific gene detected. Another factor may be due to sampling time, as we know that hemocyanin expression is downregulated at late stages of infection (46). Lastly, differences may be observed between bulk and scRNAseq studies. While bulk RNAseq is a powerful method of studying gene expression, the expression is averaged for the cells in heterogenous tissues, leading to rare cell types or transcripts being masked in the data (50). ScRNAseq enables the gene expression to be examined at the individual cell resolution (50). Thus, expression at the tissue level and individual cell level can vary leading to different results.

Consistent with previous research (12), *Fabp* expression was greater in infected shrimp compared to the control for seven clusters (HepVp1/2/4/5/6/7/8). *Fabp* likely has immune functions in shrimp as expression of *Fabp* in muscle increased in response to WSSV infection (51) and in *F. chinensis* intestine when infected with WSSV (52). Fabp typically binds to fatty acids for transport, playing a key role in lipid metabolism (53). Fabp has been demonstrated to promote VP_AHPND_. Fabp was shown to interact with the PirB toxin through two-hybrid yeast pairwise analysis (12). The involvement of Fabp in VP_AHPND_ infection was demonstrated *in vivo* through RNAi, where shrimp with *Fabp* silenced had increased survival rates following VP_AHPND_ infection (12, 54). Therefore, the pathogen appears to be exploiting this protein during infection of the hepatopancreas.

The cells from the infected group exhibited potential changes in energy metabolism. Infected_HepVp1/2/5/7 showed differential regulation of genes involved in oxidative phosphorylation. Carbohydrate and carboxylic acid metabolism are also enriched for several clusters which may relate to glucose metabolism and the TCA cycle. These pathways are implicated in mitochondrial function, which can be altered during bacterial infections to promote host immune response and by intracellular bacteria to exploit the hosts energy production system (55). Oxidative phosphorylation was associated with susceptibility to VP_AHPND_ as proteins in this pathway were upregulated in susceptible shrimp after infection but not for resistant shrimp (18). Further, changes in metabolic gene expression were more evident in susceptible shrimp compared to resistant shrimp (15, 18). As oxidative phosphorylation is upregulated in susceptible shrimp, the metabolic dysregulation may be a direct result of bacteria interference, promoting pathogenicity. Changes in energy metabolism in diverse arthropod species to bacterial infections have been detected (56, 57). However, the role of energy metabolism during infection is not understood.

In our hands, preparing single cell suspensions at later time points was challenging, likely due to the cell damage caused by the toxins. Our lab previously used bulk RNAseq to examine shrimp immune response at 1 hpi, 2 hpi, 4 hpi, and 6 hpi after exposure to recombinant PirA/B toxin and found the most differentially expressed genes at 4 hpi, so that timepoint could more closely correspond to the peak of the immune response (14). At 1 hpi and 4 hpi, MAPK signaling was detected and at 2 and 4 hpi, GTPase activity was significant. At 4 hpi, actin cytoskeleton organization and endocytosis were also significant, which may relate to the responses observed in HepVp3 cells. Examination of hemocyanin expression after immersion in 10^6^ CFU/ml of VP_AHPND_ showed that increased expression was sustained for at least 6 hpi and was down regulated at 24 and 48 hpi (46). Another study examined gene expression at 6 and 12 hpi in resistant and susceptible shrimp after immersion in 5 x 10^6^ CFU/ml and found that immune responses were sustained in the resistant families at 12 hpi (15). Therefore, the maintenance of immune response is likely affected by the genetic background of shrimp. In the current study, shrimp were infected with whole *Vibrio* cells via reverse gavage which delivers bacteria directly to the hepatopancreas. The bacteria are continuously producing toxins, and so we expect to see host responses earlier than toxin exposure studies. We also expect a faster response with reverse gavage compared to immersion studies.

## 5 Conclusions

This study investigated the single-cell transcriptome of the healthy shrimp hepatopancreas and transcriptomic responses of hepatopancreatic cells to VP_AHPND_ at single-cell resolution. Using this technology identified transcriptionally distinct cell populations and their responses to the VP_AHPND_ at 1 hour post-infection. Consistent with previous research, immune and metabolic changes occurred in the cells. Our analysis provides a basis for additional functional research, such as the properties of humoral immune proteins in host defense and energy metabolism in AHPND progression. Gene silencing or overexpression studies could further elucidate the function of specific genes, and thereby enable an understanding if expression is beneficial or detrimental to shrimp survival. Additionally, our data may be used to further characterize the hepatopancreas *in situ*.

## Data Availability

The datasets presented in this study can be found in online repositories. The names of the repository/repositories and accession number(s) can be found below: https://www.ncbi.nlm.nih.gov/geo/, GSE306674.
