# Supplementary material for: Single-cell transcriptomics of Pacific white shrimp (Litopenaeus vannamei) hepatopancreas reveal immune and metabolic responses to AHPND-causing Vibrio parahaemolyticus
Source: Front Immunol. 2026 Jan 27;17:1713369. doi: 10.3389/fimmu.2026.1713369 (PMC12902777; doi:10.3389/fimmu.2026.1713369)
Supplement: Supplementary file 1 [file DataSheet1.docx]

Supplementary Material

^
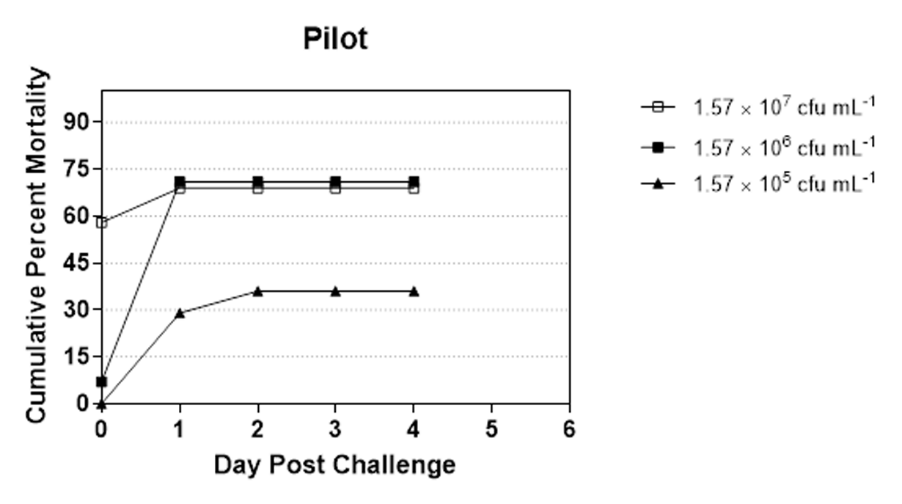
^

**Supplementary Figure 1.** Mortality of shrimp infected with low (n = 45), medium (n = 45) and high (n = 45) doses of VP_AHPND_.


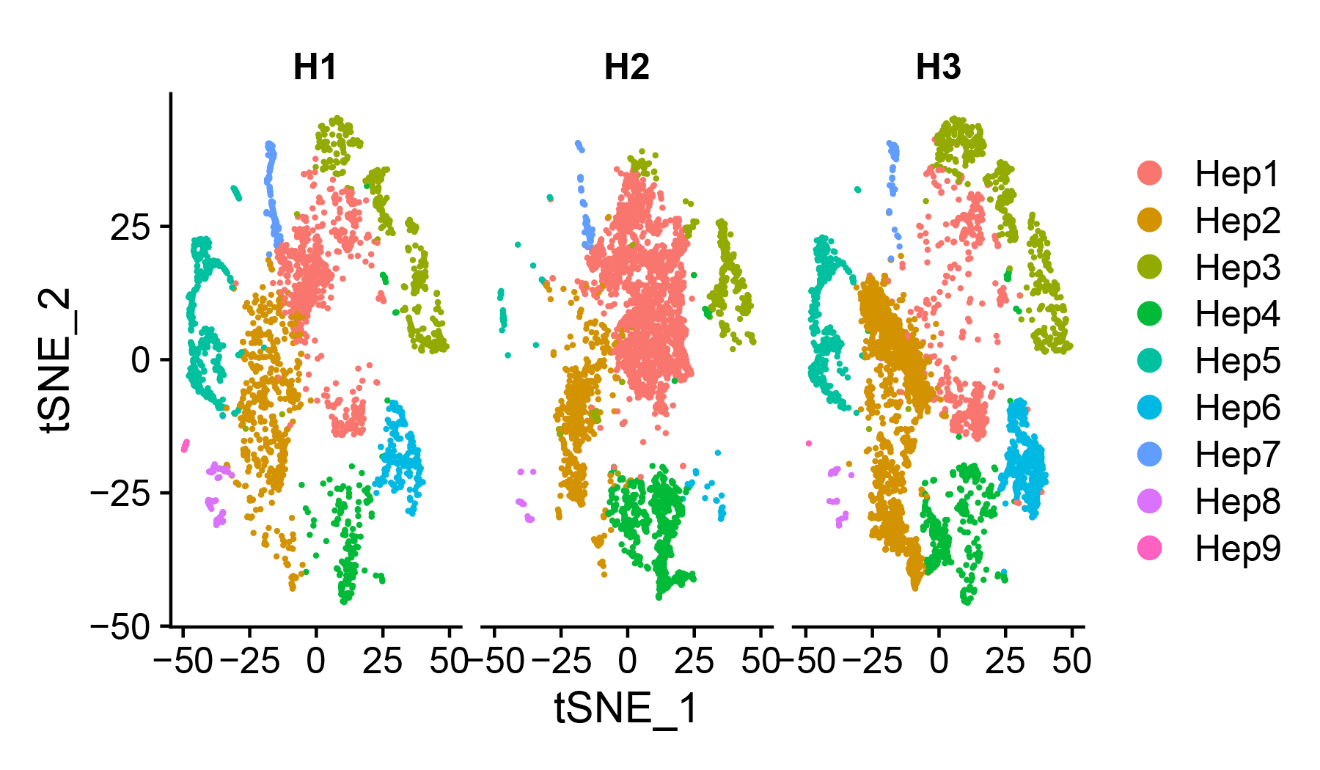
**Supplementary Figure 2.** Atlas tsne plot separated by individual sample.


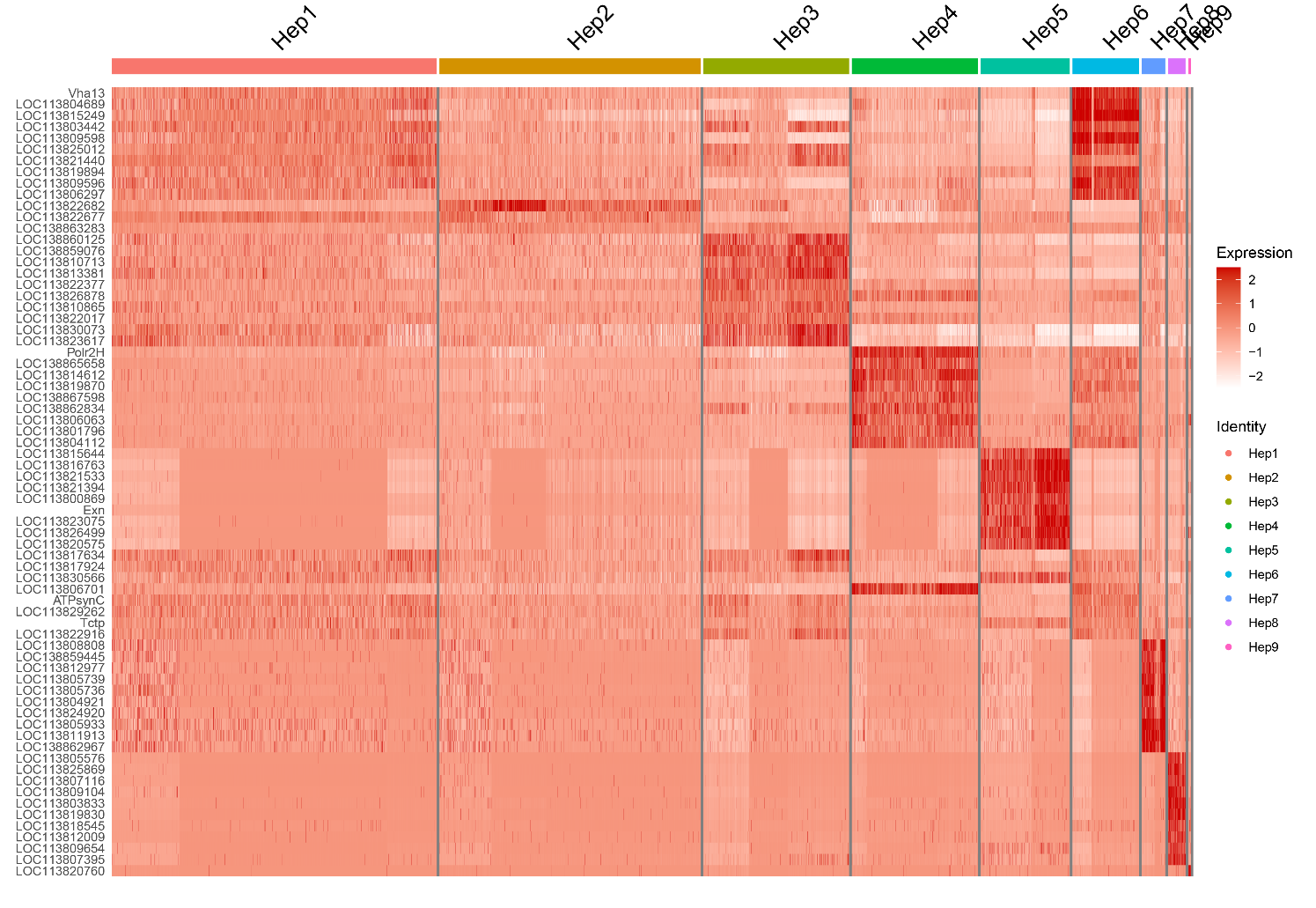


**Supplementary Figure 3.** Heatmap displaying top 10 differentially expressed genes for atlas clusters.


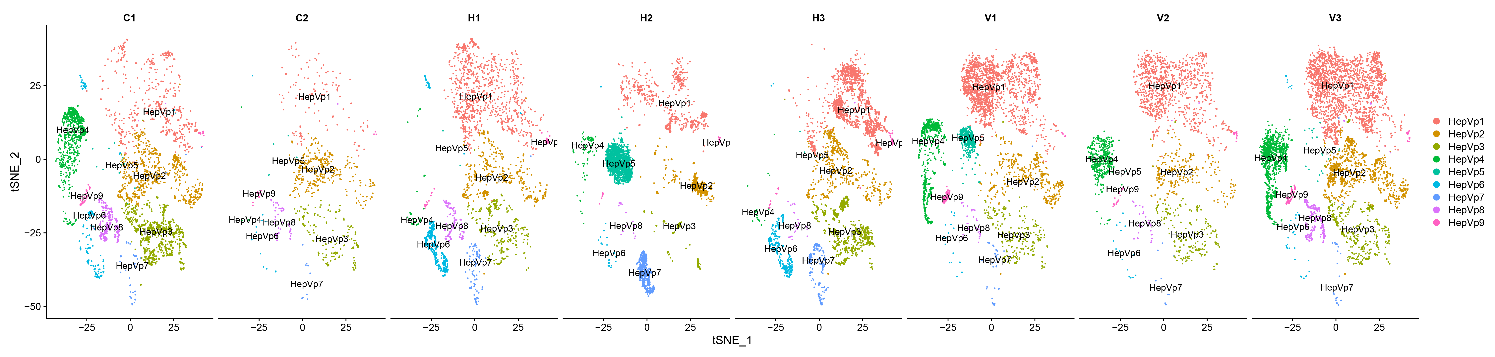


**Supplementary Figure 4.** Infection study tSNE plot separated by individual sample.


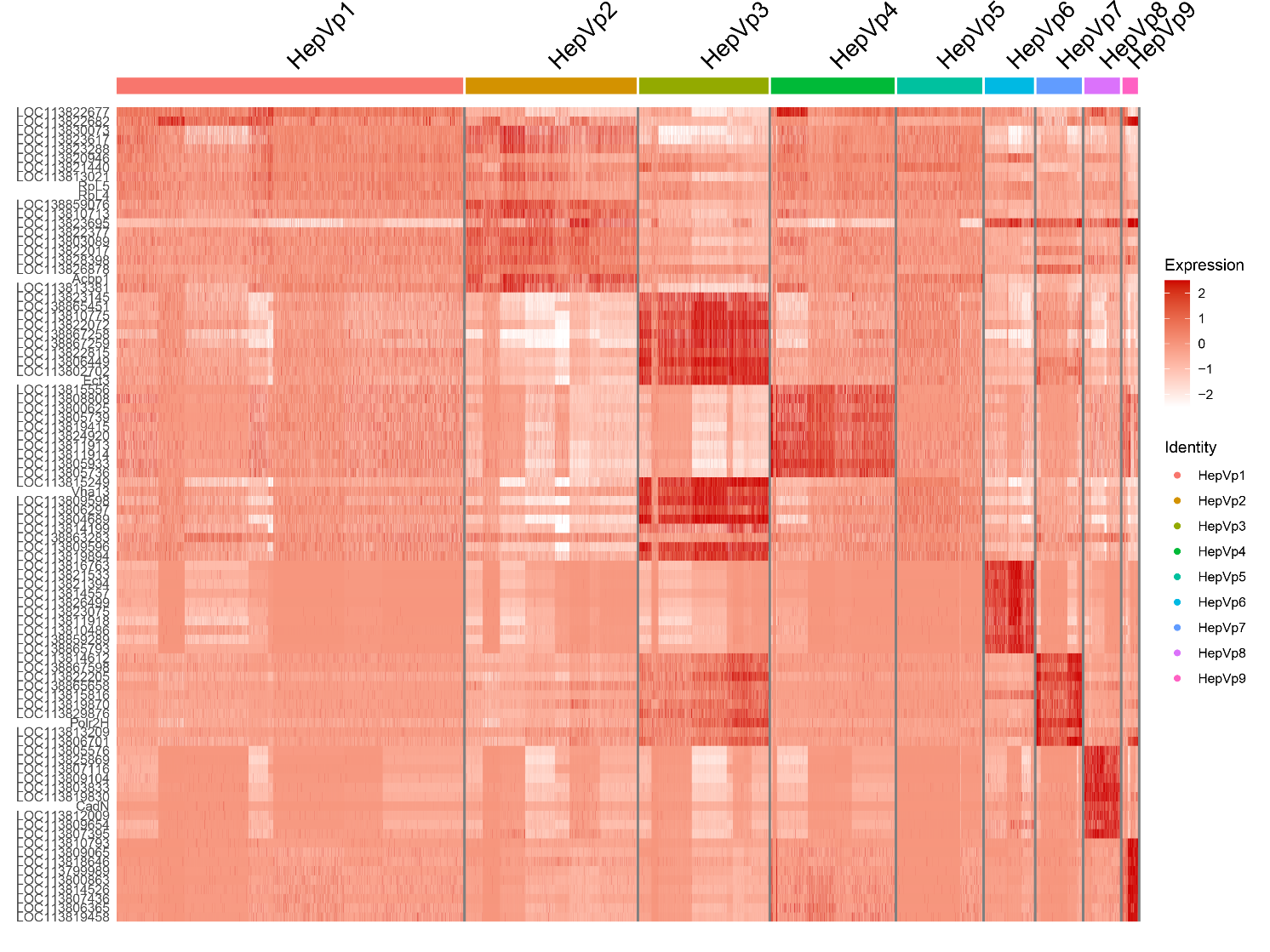


**Supplementary Figure 5.** Heatmap displaying top 10 differentially expressed genes for the infection study clusters.


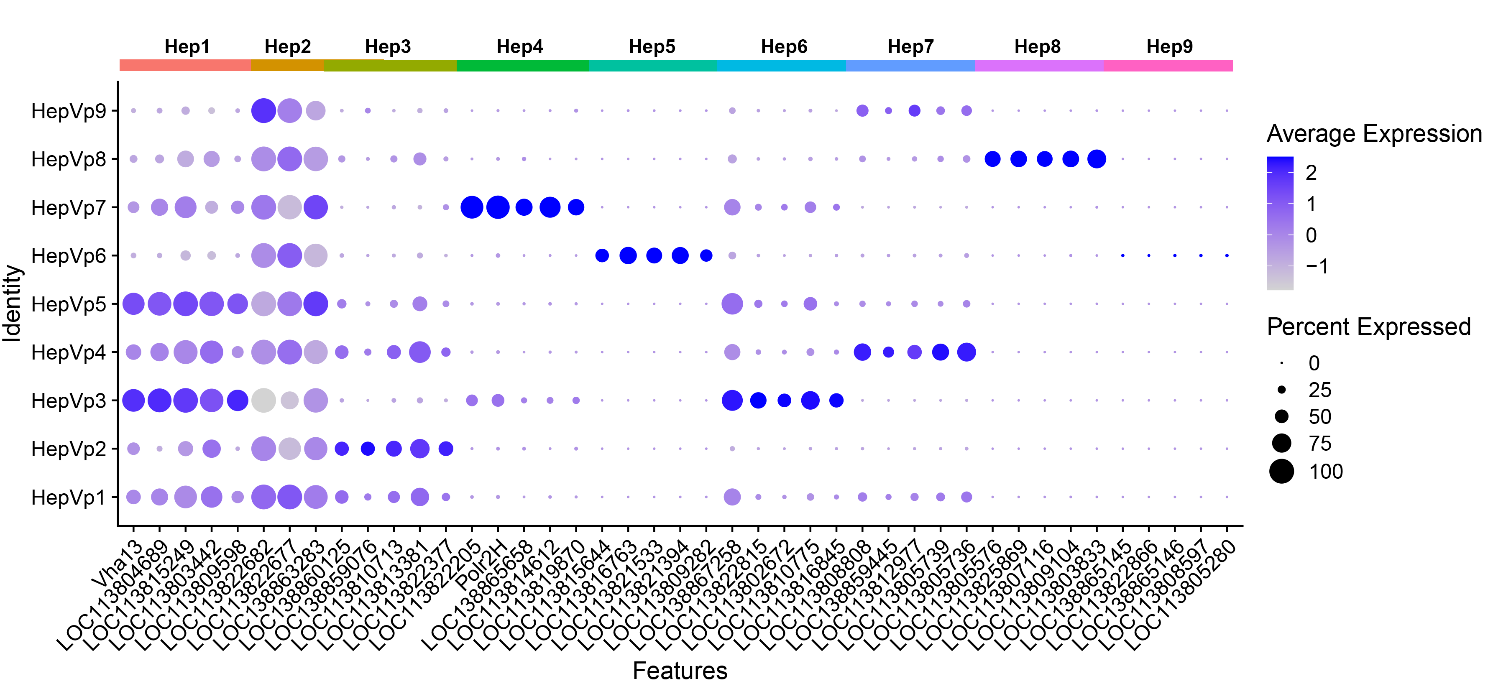


**Supplementary Figure 6.** Dot plot of top DE genes identified in the hepatopancreas atlas (Figure 2B) with the Average Expression of the genes in the *V. parahaemolyticus* infection data. This figure was used to identify matching clusters with similar expression across datasets.
